# Supplementary material for: FastqPuri: high-performance preprocessing of RNA-seq data
Source: BMC Bioinformatics. 2019 May 3;20:226. doi: 10.1186/s12859-019-2799-0 (PMC6500068; doi:10.1186/s12859-019-2799-0)
Supplement: Supplementary file 2 — Archive of FastqPuri. Archive containing all files needed to install and run FastqPuri v1.0.6. Date stamp March 22, 2019. (GZ 47,819 kb) [file 12859_2019_2799_MOESM2_ESM.gz › FastqPuri-1.0.6/html/str__manip_8c.html]

FastqPuri: src/str\_manip.c File Reference


FastqPuri


- src

Macros |
Functions

str\_manip.c File Reference

functions that do string manipulation
More...

`#include <stdio.h>`  
`#include <stdlib.h>`  
`#include <string.h>`  
`#include "str_manip.h"`

Include dependency graph for str\_manip.c:

|  |  |
| --- | --- |
| Macros | |
| #define | \_\_isascii\_c(c)   (((c) & ~0x7f) == 0) |
|  | |

|  |  |
| --- | --- |
| Functions | |
| int | str\_isascii (char \*s) |
|  | return nonzero iff all elements in the string are in the ASCII set. |
|  | |
| int | strindex (char \*s, char \*t) |
|  | returns index of t in s (start, first occurence) More... |
|  | |
| int | count\_char (char \*str, char sep) |
|  | returns the # of occurences of char c in string s |
|  | |
| int | strindexC (char \*s, char sep) |
|  | returns index of t in s (start, first occurence) More... |
|  | |
| Split | strsplit (char \*str, char sep) |
|  | Separates strings by a separator. More... |
|  | |

## Detailed Description

functions that do string manipulation

Author
:   Paula Perez paula.nosp@m.pere.nosp@m.zrubi.nosp@m.o@gm.nosp@m.ail.c.nosp@m.om

Date
:   03.08.2017

## Macro Definition Documentation

## ◆ \_\_isascii\_c

|  |  |  |  |  |  |
| --- | --- | --- | --- | --- | --- |
| #define \_\_isascii\_c | ( |  | c | ) | (((c) & ~0x7f) == 0) |

If C is a 7 bit value.

## Function Documentation

## ◆ strindex()

|  |  |  |  |
| --- | --- | --- | --- |
| int strindex | ( | char \* | *s*, |
|  |  | char \* | *t* |
|  | ) |  |  |

returns index of t in s (start, first occurence)

Parameters
:   |  |  |
    | --- | --- |
    | s | string to be checked. |
    | t | substring to be found in s. |

## ◆ strindexC()

|  |  |  |  |
| --- | --- | --- | --- |
| int strindexC | ( | char \* | *s*, |
|  |  | char | *sep* |
|  | ) |  |  |

returns index of t in s (start, first occurence)

Parameters
:   |  |  |
    | --- | --- |
    | s | string to be checked. |
    | sep | char, separator |

## ◆ strsplit()

|  |  |  |  |
| --- | --- | --- | --- |
| Split strsplit | ( | char \* | *str*, |
|  |  | char | *sep* |
|  | ) |  |  |

Separates strings by a separator.

Parameters
:   |  |  |
    | --- | --- |
    | str | input string |
    | sep | separator (char) |

Returns
:   array of strings containing the substrings in the input separated


---

Generated on Mon Mar 19 2018 23:42:01 for FastqPuri by  

 1.8.14
